# Prevalence and management of pain in lung cancer patients in northern China: A multicenter cross‐sectional study

**DOI:** 10.1111/1759-7714.14444

**Published:** 2022-05-17

**Authors:** Bo Zhang, Xingya Li, Zhiyong Ma, Shucai Zhang, Xia Song, Hongjun Gao, Liqun Gong, Yi Hu, Mengzhao Wang, Da Jiang, Cuiying Zhang, Xuedong Yuan, Baoshan Cao, Peng Zhang, Ligong Nie, Yuhui Zhang, Xiaoyan Chen, Lei Han, Wenqiang Wei, Yuankai Shi

**Affiliations:** ^1^ Department of Medical Oncology, National Cancer Center/National Clinical Research Center for Cancer/Cancer Hospital Chinese Academy of Medical Sciences & Peking Union Medical College, Beijing Key Laboratory of Clinical Study on Anticancer Molecular Targeted Drugs Beijing China; ^2^ Department of Oncology The First Affiliated Hospital of Zhengzhou University Zhengzhou China; ^3^ Department of Internal Medicine Henan Cancer Hospital Zhengzhou China; ^4^ Department of Medical Oncology, Beijing Chest Hospital Capital Medical University Beijing China; ^5^ Department of Respiratory Medicine Shanxi Provincial Cancer Hospital Taiyuan China; ^6^ Department of Oncology The Fifth Medical Center of PLA General Hospital Beijing China; ^7^ Department of Pulmonary Oncology Tianjin Medical University Cancer Institute & Hospital Tianjin China; ^8^ Department of Medical Oncology Chinese PLA General Hospital Beijing China; ^9^ Department of Respiratory Medicine Peking Union Medical College Hospital Beijing China; ^10^ Department of Medical Oncology The Fourth Hospital of Hebei Medical University and Hebei Cancer Hospital Shijiazhuang China; ^11^ Department of Medical Oncology Inner Mongolia People's Hospital Hohhot China; ^12^ Department of Oncology Peking University Third Hospital Yanqing Hospital Beijing China; ^13^ Department of Oncology Peking University Third Hospital Beijing China; ^14^ Department of Thoracic Surgery General Hospital of Tianjin Medical University Tianjin China; ^15^ Department of Respiratory Peking University First Hospital Beijing China; ^16^ Department of Respiratory, Beijing Chaoyang Hospital Capital Medical University Beijing China; ^17^ Department of Medical Oncology Beijing Shunyi Hospital Beijing China; ^18^ Department of Medical Oncology People's Hospital of Beijing Daxing District Beijing China; ^19^ Department of Epidemiology, National Cancer Center/National Clinical Research Center for Cancer/Cancer Hospital Chinese Academy of Medical Sciences & Peking Union Medical College Beijing China

**Keywords:** cancer pain, lung cancer, pain management, prevalence, survey

## Abstract

**Background:**

Pain is a fearful yet common symptom among lung cancer patients. This multicenter, cross‐sectional study was conducted to examine the current status of pain prevalence and management in lung cancer patients in northern China.

**Methods:**

A total of 18 hospitals across northern China were selected. Patients with primary lung cancer who visited the outpatient clinic or were admitted in the wards on a preplanned day were invited to complete a questionnaire. Meanwhile, physicians who had experience of treating primary lung cancer patients were also surveyed.

**Results:**

A total of 533 patients and 197 physicians provided valid responses to the survey, of which 45.4% (242/533) of patients reported pain during the course of disease and 24.2% (129/533) of patients had experienced pain within the past 24 h. The mean average pain intensity by the brief pain inventory was 3.47 ± 1.55. The binary logistic regression analysis showed female gender and stage IV disease were significantly associated with the presence of pain. A total of 74.4% (96/129) of patients reporting pain within 24 h were taking analgesics. The most common reason for patients not using analgesics was that the pain was tolerable (48.2%), while the most common barriers to prescribing opioids as reported by physicians were fear of adverse reactions (43.7%) and fear of addiction (43.1%).

**Conclusion:**

Despite recognition of the importance of pain control by most physicians and an improvement in cancer pain management, inadequate treatment of cancer pain still exists in lung cancer patients in northern China. High‐quality pain education for both patients and physicians is needed in the future.

## Graphical abstract

We conducted a multicenter, cross‐sectional study in 18 hospitals across northern China to evaluate the prevalence and management of pain in lung cancer patients. Among the 533 patients surveyed, 45.4% (242/533) suffered pain in the course of their disease. Meanwhile, 24.2% (129/533) experienced pain within 24 h, only 74.4% (96/129) of whom were taking analgesics, suggesting inadequate treatment of cancer pain. There is still need for high‐quality pain education for patients and physicians.

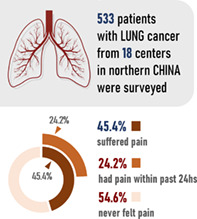

## INTRODUCTION

Lung cancer is one of the leading cancer entities and mortality worldwide, with an estimated 2.2 million new cancer cases and 1.8 million deaths in 2020.^1^ Despite advances in the treatment of lung cancer and the improvement in palliative care in the past decades, pain remains one of the most fearful and burdensome symptoms in lung cancer patients. The prevalence of pain in lung cancer patients ranked the third among all malignancies, following head/neck and gastrointestinal cancers.^2^ Undertreatment of cancer pain is associated with physical and psychological consequences, which seriously affects the quality of life of patients.^3^ Therefore, pain management is considered an essential component and one of the primary goals of cancer treatment.^4^

Although the influences of pain on clinical outcomes have attracted growing attention, undermanaged pain is still common in cancer patients. The major barriers to adequate pain control involve the physicians, patients, and healthcare system. Common professional‐related barriers include poor assessment of pain, inadequate knowledge and skill, and reluctance to prescribe opioids. From the patients' perspective, the barriers include cognitive factors (concern regarding disease progression or addiction), affective factors (psychological distress), and adherence to analgesic regimens.^5^ Understanding of the underlying professional or cognitive barriers, as well as the awareness of the current status of pain management, have great significance in designing effective educational interventions to improve cancer pain management.

At present, data are lacking on the status of cancer‐related pain in Chinese lung cancer patients, including the prevalence, evaluation and management of cancer‐related pain, the reasons for undermedication, and patients' perception and physicians' knowledge of pain control. Although much effort has been devoted to uncover the underlying association between clinical‐pathological characteristics and treatment outcomes of lung cancer patients, few studies have investigated the possible factors affecting the incidence of lung cancer‐related pain, such as age, gender, stage of cancer and history of smoking. Therefore, we initiated a cross‐sectional study by questionnaire survey of participating patients and physicians from multiple centers in northern China, to evaluate the prevalence and characteristics of lung cancer‐related pain, the correlation between pain and clinicopathological factors, and the attitudes of patients and physicians towards pain control.

## METHODS

### Procedures and participants

A total of 18 participating centers in northern China were selected from January 16, 2018 to January 29, 2018, based on convenience sampling from two municipalities (Beijing and Tianjin), three provinces (Hebei, Henan and Shanxi), and one autonomous region (Inner Mongolia). Each center performed the study on a single, prespecified day determined by randomization. Two sets of questionnaires were designed for patients and physicians, respectively, and distributed in the clinics and wards of relevant departments that treated lung cancer patients (including department of medical oncology, department of respiratory medicine, and department of thoracic surgery). The inclusion criteria of patients were: (1) pathologically diagnosed with lung cancer, (2) visited the outpatient clinic or admitted in the relevant wards, (3) had sufficient knowledge of spoken and written Chinese, and (4) consented to participate in the study. Physicians who had experience of treating primary lung cancer were surveyed.

Questionnaires for patients included demographic data, presence and intensity of cancer pain, details of management, attitudes toward usage of analgesics, level of satisfaction with pain control, and their access to cancer pain education. The intensity of cancer‐related pain was evaluated with the brief pain inventory–short form (BPI‐sf). Questionnaires for physicians focused on previous education with pain control, and routine practice with assessment, management, follow‐up, and patient education of cancer pain. The responses to the questionnaire were collected in the form of face to face interview.

### Statistical analysis

Patient characteristics were summarized using absolute numbers and percentages. Normal distribution of data is presented as mean ± standard deviation (SD). To identify factors significantly associated with the presence of cancer‐related pain, clinicopathological characteristics including gender, age, pathological type of lung cancer, smoking status and TNM stage were first categorized and examined by *χ* ^2^ test. A binary logistic regression model was established, using the presence of pain as the dependent variable and the categorized clinicopathological characteristics as the independent variables. All probability values are two‐sided, and *p* < 0.05 was considered statistically significant. All analyses were performed using SPSS software, version 22.0 (SPSS Inc).

## RESULTS

### Characteristics of the patients and physicians surveyed

The study questionnaires were collected from 533 patients and 197 physicians who provided complete and valid responses. The mean age of the patients was 59.1 years (range: 23–84), among whom 354 (66.4%) were male. A total of 166 patients (31.1%) were surveyed in the outpatient clinic, 367 patients (68.9%) were from the inpatient wards. Most patients had adenocarcinoma (52.5%, 280/533), and 310 patients (58.2%) presented with stage IV disease. Most of the physicians were medical oncologists (67.5%, 133/197). The demographic characteristics of the patients and physicians are summarized in Tables 1 and 2, respectively.

**TABLE 1:** Demographic characteristics of patients (*N* = 533)

Patient characteristics	Value
Age (years), mean ± SD	59.1 ± 10.8
Gender, *N* (%)	
Female	179 (33.6)
Male	354 (66.4)
Source of patients, *N* (%)	
Outpatient clinic	167 (31.3)
Inpatient wards	366 (68.7)
Histological subtype, *N* (%)	
Small cell lung cancer	107 (20.1)
Adenocarcinoma	280 (52.5)
Squamous cell carcinoma	97 (18.2)
Large cell carcinoma	4 (0.8)
Not specified	45 (8.4)
TNM staging, *N* (%)	
I	46 (8.6)
II	35 (6.6)
III	113 (21.2)
IV	310 (58.2)
Missing information	29 (5.4)
Duration of pain, *N* (%)	
Experienced pain within 24 h	129 (24.2)
No pain within 24 h, but experienced pain previously	113 (21.2)
Never felt pain	291 (54.6)

Abbreviation: SD, standard deviation.

**TABLE 2:** Demographic characteristics of physicians (*N* = 197)

Physician characteristics	Value
Professional title, *N* (%)	
Directors and vice‐directors	66 (33.5)
Attending physician, residents, and others	131 (66.5)
Education background, *N* (%)	
Doctor/Master of medicine	164 (83.2)
Bachelor of medicine and others	33 (16.8)
Subspecialty, *N* (%)	
Medical oncology	133 (67.5)
Thoracic surgery	31 (15.7)
Respiratory medicine	16 (8.1)
Others	17 (8.6)

### Prevalence, body location, and severity of cancer pain

Among the 533 lung cancer patients, 242 patients (45.4%) experienced pain during the course of disease, 291 patients did not. A total of 125 patients (51.7%) experienced pain in one body site, 76 patients (31.4%) had pain in two body sites. The most common sites of pain were chest in 117 patients (48.3%), back in 81 patients (33.5%), and waist and abdomen in 39 patients (16.1%). The cancer pain was most frequently described as dull (18.2%, 44/242) or swelling pain (11.6%, 28/242). A total of 129 patients (24.2%) experienced pain within 24 h before survey, the mean average pain intensity (API) was 3.47 ± 1.55, and API was severe (7–10 points) in seven patients, moderate (4–6 points) in 46 patients, and mild (1–3 points) in 76 patients. The major characteristics of pain are summarized in Table 3. The prevalence of pain in patients with stage IV disease and bone metastasis was also calculated. Among 310 patients with stage IV disease, 168 patients (54.2%) experienced cancer pain, 87 patients (28.1%) experienced pain within 24 h before the survey. Among the 144 patients with known bone metastasis, 100 (69.4%) had cancer pain, and 56 (38.9%) experienced pain within the last 24 h.

**TABLE 3:** Characteristics and management of pain

Characteristics of pain	Value
Presence of pain (*N* = 533), *N* (%)	
Experienced pain in the course of lung cancer	242 (45.4)
Pain within 24 h	129 (24.2)
No pain	291 (54.6)
No. of body sites with pain (*N* = 242), *N* (%)	
1	125 (51.7)
2	76 (31.4)
≥3	41 (16.9)
Body location of pain (*N* = 242), *N* (%)	
Chest	117 (48.4)
Back	81 (33.5)
Waist and abdomen	39 (16.1)
Head and neck	23 (9.5)
Limbs	4 (1.7)
BPI‐sf pain intensity (*N* = 129), mean ± SD	
Worst pain intensity	5.12 ± 2.18
Average pain intensity	3.47 ± 1.56
Least pain intensity	2.09 ± 1.69
Pain right now	2.90 ± 2.02
BPI‐sf API categorization (*N* = 129), *N* (%)	
Mild	76 (58.9)
Moderate	46 (35.7)
Severe	7 (5.4)
Description of pain (*N* = 242), *N* (%)	
Dull	44 (18.2)
Swelling	28 (11.6)
Stabbing	21 (8.7)
Treatment of pain (*N* = 242), *N* (%)	
No treatment	114 (47.1)
Taking analgesics	128 (52.9)
NSAIDs	12 (9.4)
Opioids	108 (84.4)
Others	8 (6.3)
Missing information	5 (3.9)
Satisfaction with pain relief (*N* = 242), *N* (%)	
Satisfied	160 (66.1)
Neutral	44 (18.2)
Unsatisfied	4 (1.7)
Missing information	34 (14.0)

Abbreviations: API, average pain intensity; BPI‐sf, brief pain inventory–short form; NSAIDs, nonsteroidal anti‐inflammatory drugs; SD, standard deviation.

### Patient characteristics in relation to the presence of pain

Patients were divided into two groups based on whether they experienced pain: patients with pain group (*n* = 242) and patients without pain group (*n* = 291). The association of pain with the major clinicopathological characteristics of patients are listed in Table 4. The results showed gender and TNM stage were significantly associated with the presence of cancer pain (gender, *p* = 0.020; TNM stage, *p* < 0.001). We then identified that gender (*p* = 0.040) and TNM stage (*p* = 0.007) significantly affected the presence of pain using a binary logistic regression model. As shown in Table 5, female patients were 1.531 times more likely to experience pain than male patients, while patients with stage IV disease were 2.653 times more likely to experience pain than patients with stage I disease.

**TABLE 4:** Association of pain with patient characteristics

Characteristics	Patients with pain (*n* = 242)	Patients without pain (*n* = 291)	*p‐*value
Age (years), *N* (%)			0.472
<50	46 (19.0)	59 (20.3)	
50–60	68 (28.1)	65 (22.3)	
60–70	86 (35.5)	116 (39.9)	
≥70	42 (17.4)	51 (17.5)	
Gender, *N* (%)			0.021
Female	94 (38.8)	85 (29.2)	
Male	148 (61.2)	206 (70.8)	
Histological subtype, *N* (%)			0.218
Small cell lung cancer	45 (18.6)	62 (21.3)	
Adenocarcinoma	129 (53.3)	151 (51.9)	
Squamous cell carcinoma	40 (16.5)	57 (19.6)	
Large cell carcinoma	1 (0.4)	3 (1.0)	
Not specified	27 (11.2)	18 (6.2)	
TNM stage, *N* (%)			<0.001
I	18 (7.4)	28 (9.6)	
II	11 (4.6)	24 (8.3)	
III	31 (12.8)	82 (28.2)	
IV	168 (69.4)	142 (48.8)	
Missing information	14 (5. 8)	15 (5.2)	
Smoking status, *N* (%)			0.481
Never smokers	98 (40.5)	127 (43.6)	
Smokers	144 (59.5)	164 (56.4)	

**TABLE 5:** Binary logistic regression model for cancer pain

Variables	*β*	SE	Wald *χ* ^2^ value	*p*‐value	OR value	95% CI
Constant	−0.914	0.520	3.087	0.079	0.401	
Gender						
Female	0.426	0.208	4.207	0.040	1.531	1.019–2.300
TNM stage			27.539	0.000		
II	0.076	0.516	0.022	0.883	1.079	0.392–2.968
III	−0.184	0.411	0.201	0.654	0.832	0.372–1.862
IV	0.976	0.364	7.192	0.007	2.653	1.300–5.414

Abbreviations: OR, odds ratio; SE, standard error.

### Patients' pain relief and attitude to pain

In the patients' survey, 95.0% patients (230/242) reported pain to physicians, and 83.1% patients (201/242) responded that their physicians would actively inquire and assess pain. A total of 52.9% patients (128/242) were taking analgesics for cancer pain, 44.6% patients (108/242) were receiving opioids. Specifically, the percentage of patients taking analgesics was 74.4% (*n* = 96) in the 129 patients who reported pain within the past 24 h, and 28.3% (*n* = 32) in the 113 patients who had experienced pain but not within the past 24 h. Notably, among the 33 patients who had pain within 24 h but were not taking analgesics, 17 reported the worst pain intensity of 4–6. The most common reason for not taking analgesics from the patients' responses was “can tolerate pain on own” (48.2%, 55/114). Seventy‐three out of the 108 patients using opioids reported constipation as one of the side effects, and 66 of them reported that physicians prescribed laxatives to relieve constipation.

Eighty‐seven (77.0%) out of the 113 patients who had experienced pain but not within the past 24 h were satisfied with their pain control, 18 patients (15.9%) were neutral, while eight patients did not provide an answer. Out of the 129 patients who had pain within the past 24 h, 73 (56.6%) were satisfied, 26 (20.2%) were neutral, 26 (20.2%) did not respond to the question, and four (3.1%) were dissatisfied. Of these, two patients reported the analgesics were not effective, whereas the other two thought their pain had been overlooked by physicians.

### Physicians’ knowledge of pain control

A total of 85.8% of physicians (169/197) reported that pain management was included as part of medical education, and 94.9% of physicians (187/197) had received continuing medical education (CME) regarding cancer pain management. A total of 59.4% of physicians (117/197) agreed to carry out at least 5 h of cancer pain CME every year. However, only 46.2% of physicians (91/197) actually received over 5 h of cancer pain CME during the past year, and 88.8% (175/197) of physicians reported that they referred to mainstream guidelines for cancer pain management, including the World Health Organization Cancer Pain Relief (90.9%, 159/175), the Chinese Guidelines for the diagnosis and treatment of cancer pain (61.1%, 107/175), and the National Comprehensive Cancer Network guidelines for adult cancer pain (58.9%, 103/175).

### Physicians' assessment and management of pain

A total of 84.7% of physicians (167/197) conducted pain‐screening routinely, while 9.6% (19/197) only did it “sometimes.” Numeric rating scale (NRS) was most commonly used for pain assessment (74.1%, 146/197), followed by visual analog scale (VAS), verbal rating scale (VRS) and BPI. A total of 18.3% (36/197) of physicians could control patients' pain within 1 day upon appropriate medication, while 72.6% (143/197) of physicians required 2–3 days, and 96.4% (190/197) of physicians were satisfied with cancer pain management. A total of 89.3% (176/197) of physicians agreed that opioids were the first‐line analgesics in cancer patients with moderate–severe pain, and 85.8% (169/197) preferred oral opioids (except the setting when oral administration was impossible or impractical). The most common barriers to prescribing opioids as reported by physicians were patients' fear of adverse reactions (43.7%, 86/197), patients' fear of addiction (43.1%, 85/197), and worry by physicians that adverse reactions may not be tolerated (32.0%, 63/197, Table 6).

**TABLE 6:** Barriers for opioids prescription as reported by physicians

Barriers	*N* (%)
Patients' fear of adverse reactions	86 (43.7)
Patients' fear of addiction	85 (43.1)
Physicians' worry that adverse reactions may not be tolerated	63 (32.0)
Complicated procedures for prescribing opioids	42 (21.3)
Complicated opioid‐to‐opioid conversions calculations	27 (13.7)
Patient unable to afford opioids	21 (10.7)
Difficulties in managing adverse reactions	20 (10.2)
Others	3 (1.5)
No barriers reported	42 (21.3)

A total of 97.0% (191/197) of physicians declared that they provided education on pain management to patients. The content of patient education included general knowledge of pain, assessment and treatment options of pain, and management of adverse reactions with analgesics. The frequencies of follow‐up with outcomes of pain relief were once every week for 80 physicians (40.6%), once every month for 76 physicians (38.6%), and once every 1–3 months for 21 (10.7%) physicians. Seven physicians (3.6%) reported the follow‐up interval of more than 3 months, while 13 physicians (6.6%) did not follow up their pain control outcomes.

## DISCUSSION

Pain is one of the most common symptoms in cancer patients, and it can be secondary to malignant tumors or treatment, which seriously affects the quality of life of patients. Pain has been thought to affect survival in cancer patients, although the mechanism remains unknown. A meta‐analysis demonstrated that pain was a prognostic factor for shorter survival, in addition to demographic and clinical factors.^6^ Pain was identified as an independent prognostic factor for overall survival of prostate cancer, and might have similar prognostic value in breast, colorectal, or lung cancers.^7^ However, successful management of cancer pain still presents a considerable challenge. In China, lung cancer is the most common incident cancer and the leading cause of cancer death, creating a huge disease burden.^8^ The current status of pain management in lung cancer patients may provide a general picture of cancer pain control, and guide the future pain relief actions in China.

In the present cross‐sectional study, one important finding was that 45.4% of lung cancer patients reported suffering from pain, which may represent a period prevalence. Meanwhile, 24.2% of patients had pain within 24 h, this was regarded as a point prevalence, reflecting patients immediately in need of appropriate pain control. Two systemic reviews conducted in the western hemisphere have reported that the overall prevalence of pain in lung cancer patients was 47% and 55% in 2004 and 2007, respectively.^2^, ^9^ The percentage of patients with a negative pain management index (PMI), representative of the degree of undertreatment of pain, was 67 in a study conducted in the Chinese population in 1996.^10^ In comparison, the mean percentage of a negative PMI in the United States and Europe was 39.1 and 40.3, respectively, showing a gap in cancer pain control between western countries and China in the past. In a Chinese nationwide survey, which was conducted in 29 provinces and included 1555 patients in 1999, cancer‐related pain occurred in 61.6% of patients.^11^ In the present study, most patients had mild or moderate pain within 24 h, with a mean API of 3.47. Apart from the influences of the heterogeneities in a study setting and patient selection, one possible reason for the lower prevalence could be the initiation of the Good Pain Management program by the Ministry of Health of the People's Republic of China in March 2011. The nationwide program involved training and education for cancer pain treatment for both physicians and patients to standardize the diagnosis and treatment of cancer pain, improve the quality of life for patients with cancer, and safeguard the quality and safety of health care services.^12^

In the present study, chest was the most common site of pain, which could be the result of pleural involvement or bone metastasis. Moreover, it has been found that roughly half of patients who underwent thoracic surgery reported clinically significant pain, while its management was not sufficient to result in acceptable outcomes.^13^ In an Italian observational study, the incidence of chronic post‐surgical pain at 12 months was 35.9%, 11.8% and 2.5% for mild, moderate and severe pain, respectively.^14^ We were unable to examine the causal effect of thoracic surgery on cancer pain in our present study because detailed information regarding history of surgery was not collected, and further studies are warranted for postoperative pain control in lung cancer patients.

Risk factors for the development of pain had been investigated in malignancies other than lung cancer. For instance, in a previous study, higher BMI, fewer schooling years, not smoking, chemotherapy, hormone therapy, and radiotherapy were significantly associated with higher odds for the development of chronic pain in breast cancer patients.^15^ There was limited information regarding predictors of pain among lung cancer patients, with several studies reporting inconsistent findings about influencing factors of chronic post‐thoracotomy pain.^16^, ^17^, ^18^, ^19^ Our study revealed that, compared with patients with stage I disease, patients with stage IV disease were at significantly increased risk of pain, while patients with stage II or III disease were not. Our findings implied that direct invasion of distant metastasis might be an important contributing factor of pain in lung cancer patients. Meanwhile, it was found that female patients were more likely to suffer pain than males, which was consistent with previous studies.^20^, ^21^ Multiple biopsychosocial mechanisms have been proposed to explain this sex difference, including sex hormones, endogenous opioid function, genetic factors, and pain coping.^21^ However, a clear elucidation has not been established, and sex‐specific tailoring of pain control, including cancer pain, was not supported by the currently available evidence.

Lack of time and knowledge about cancer pain assessment and management in healthcare providers are the leading causes for inadequate pain control among oncology patients.^22^ The results in our study from both physicians and patients revealed that screening and assessment of pain were performed in most cases. However, 33 (25.6%) patients who had pain within the past 24 h were not taking analgesics, indicating the possibility of inadequate treatment. Interestingly, over half of patients who had pain within the past 24 h were satisfied with pain control, including those who were not using analgesics. These results suggested that patient satisfaction with pain control might not completely correlate with the presence or severity of pain, and similar findings were observed in a study involving in patients receiving opioid therapy in an acute care institution.^23^

Physicians' considerations when prescribing opioids to patients were fear of addiction and adverse reactions, and these findings are consistent with previous studies of the major barriers of cancer pain management.^5^, ^24^ Educational interventions for patients have proven efficacious in improving pain outcomes in cancer patients.^25^, ^26^, ^27^ Acknowledging the barriers, physicians may help their lung cancer patients better understand the benefits of eliminating pain rather than bearing it, in addition to providing instructions regarding opioid therapy‐related adverse effects. The physicians appear to have recognized the importance of pain education, with most of them receiving cancer pain‐related CME each year. Proper patient follow‐up and continuation of counselling with telephone after outpatient clinic visit or discharge have been found to improve quality of life in cancer patients.^28^, ^29^ In our present study, the majority of physicians followed up their patients and offered additional support concerning pain management.

Our study had limitations. The cross‐sectional design was incapable of showing the evolution of lung cancer pain prevalence and management over time. Additionally, most centers were tertiary hospitals with relatively high standards of care, and convenience sampling might have introduced selection bias.

In conclusion, despite the recognition of the importance of pain control by most physicians and an improvement in cancer pain management, inadequate treatment of cancer pain still exists in lung cancer patients in northern China. As the largest epidemiology study evaluating pain in Chinese lung cancer patients to date, this study has provided some key facts to understand the current status of pain management in a common malignancy. In the future, efforts should be made to improve the quality of pain management education for lung cancer patients and physicians.

## CONFLICT OF INTEREST

The authors declare that there are no conflicts of interest.
